# Catheter ablation of para-hisian premature ventricular contractions using electroanatomical mapping: Approaches and pitfalls

**DOI:** 10.14744/nci.2020.47897

**Published:** 2022-05-11

**Authors:** Gokhan Aksan, Osman Can Yontar, Ahmet Yanik, Guney Erdogan, Ugur Arslan

**Affiliations:** Department of Cardiology, Samsun Training and Research Hospital, Samsun, Turkiye

**Keywords:** Para-hisian, premature ventricular contraction, radiofrequency ablation

## Abstract

A 58-year-old female patient presented at cardiology outpatient clinic with palpitation. The 12-lead electrocardiography on admission revealed monomorphic bigeminy premature ventricular contractions (PVCs) showed a left bundle-branch block configuration, monophasic R wave in lead I and aVL and precordial transition in V3 lead. Cardiac electrophysiological study was performed to patient. Activation mapping guided by three-dimensional electroanatomic system was done. The earliest ventricular activation was observed in the para-hisian region with the largest His potential (0.6 mV) during PVC. Due to the risk of atrioventricular (AV) block, radiofrequency (RF) ablation was planned to the region, where the His potential amplitude was lower (0.2 mV), the AV ratio was <1, and ventricular activation preceded the QRS onset by 37 ms. Subsequently, irrigated RF current was delivered in the distal His region with power starting at 15 W after PVC was suppressed, RF delivery was applied for a total of 90 s with gradually increasing power to 25 W. After ablation, under isoproterenol infusion, burst pacing from the right ventricle no PVCs/VTs was observed. A gradual RF energy application, a detailed activation mapping, and the distance from the largest His potential increase the likelihood of success in para-hisian PVC ablation.

Most idiopathic premature ventricular contractions (PVCs) originate from the right or left ventricular outflow tracts and aortic sinuses of Valsalva; however, it may also arise from the para-hisian region, even though it is rare [1]. The radiofrequency (RF) catheter ablation for PVC treatment is preferred for symptomatic patients resistant to medical treatment [2]. RF ablation of PVCs from para-hisian region can be frequently challenging due to high risk of iatrogenic atrioventricular (AV) block, but also have lower long-term success rate than other locations [3].

Here, we reported a case of successful catheter ablation of PVCs arising from the para-hisian region with a three-dimensional (3D) electroanatomic mapping system, and ablation techniques for such cases.

## CASE REPORT

**Figure 1:**
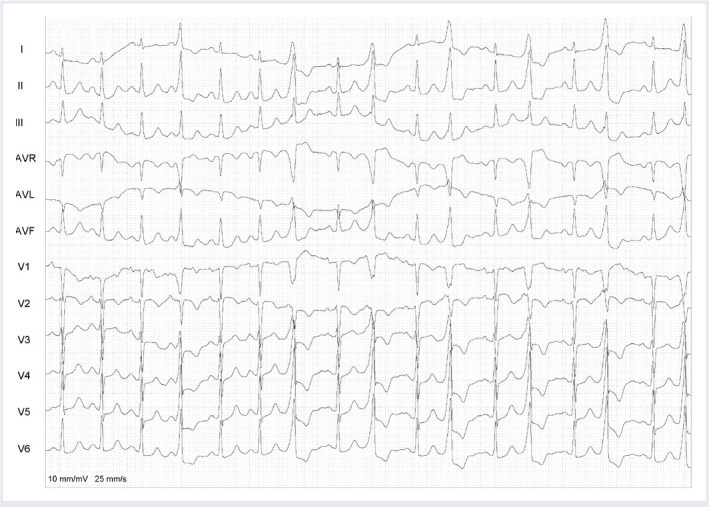
A 12-lead ECG at 10 mm/mV 25 mm/s of the patients on admission. ECG: Electrocardiogram.

A 58-year-old female patient presented at the cardiology outpatient clinic with palpitation and dyspnea. The patient’s 12-lead electrocardiography (ECG) on admission showed monomorphic bigeminy PVCs. In addition, PVC showed a left bundle-branch block configuration, monophasic R wave in lead I and aVL, and precordial transition in V3 lead (Fig. 1). His symptomatic PVCs were refractory to class I-III antiarrhythmic agents (Propafenone 2 × 150 mgr and amiodarone 2 × 200 mgr, respectively). Echocardiographic measurements were normal. The 24-h ambulatory ECG monitoring revealed frequent monomorphic bigeminy and trigeminy PVCs (28.082 beats; 25.3% of total beats). The patient stated that there were palpitations and dyspnea during the 24-h ambulatory ECG monitoring. This finding proved that clinical PVCs are symptomatic. No significant coronary artery stenosis was observed at coronary angiography performed for coronary ischemia.

**Figure 2:**
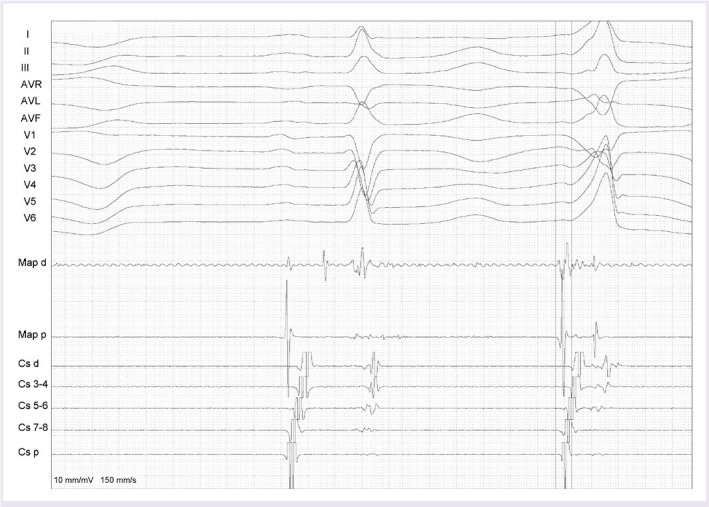
The endocardial electrograms at the para-hisian ablation site exhibiting the earliest ventricular activation during the PVCs. Large His-bundle electrogram was recorded from the Map-d during sinus rhythm and the earliest ventricular activation preceding the QRS by 41 ms was observed during PVC. PVC: Premature ventricular contraction, Map-d: Bipolar recording from the distal electrode of the ablation catheter.

After informed consent was obtained from the patient, a cardiac electrophysiological study was performed under a fasting non-sedated state and antiarrhythmic agents had been discontinued for at least five half-lives before the study. For mapping and pacing, a quadripolar catheter was positioned via right femoral vein at the right ventricular (RV) apex and a deflectable decapolar catheter in the coronary sinus. Mapping and pacing were performed using a 3.5 mm open-irrigated-tip catheter (ThermoCool, Biosense Webster, Diamond Bar, CA, USA). Activation mapping guided by three-dimensional electroanatomic system was performed to identify the earliest site of ventricular activation the PVCs. The activation time was measured from the onset of the bipolar electrogram from the distal bipole of the mapping catheter to the earliest onset of the QRS complex in any of the 12 ECG leads. During the PVC, the earliest ventricular activation was identified at the His-bundle region, where a high amplitude His-bundle electrogram could be recorded during the sinus rhythm. PVC activation preceded QRS onset by 41 ms was recorded on the bipolar recording with QS pattern on the unipolar recording and there was a 0.6 mV His potential (Fig. 2).

**Figure 3:**
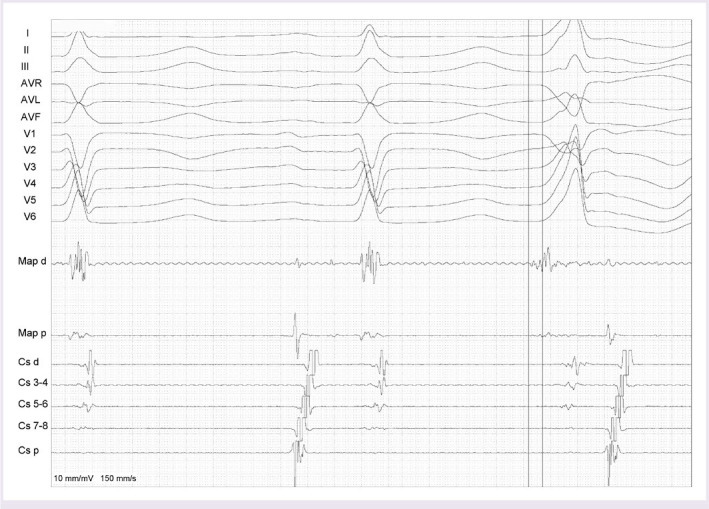
Distal His-bundle electrogram was recorded from the Map-d during sinus rhythm and the ventricular activation preceding the QRS by 37 ms was observed during PVC at the successful ablation site. PVC: Premature ventricular contraction; Map-d: Bipolar recording from the distal electrode of the ablation catheter.

**Figure 4:**
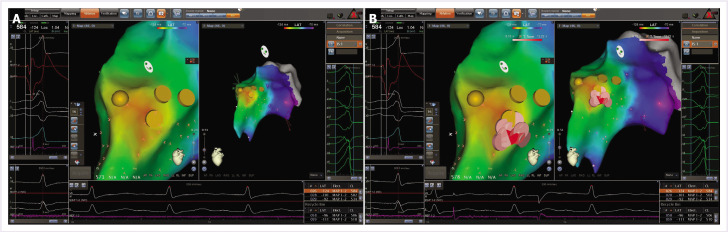
Simultaneous anatomical and activation mapping using a three-dimensional electroanatomic mapping system at the para-hisian region. Ventricular activation preceding the QRS by 37 ms was observed during PVC at the successful ablation site. PVC: Premature ventricular contraction.

**Figure 5:**
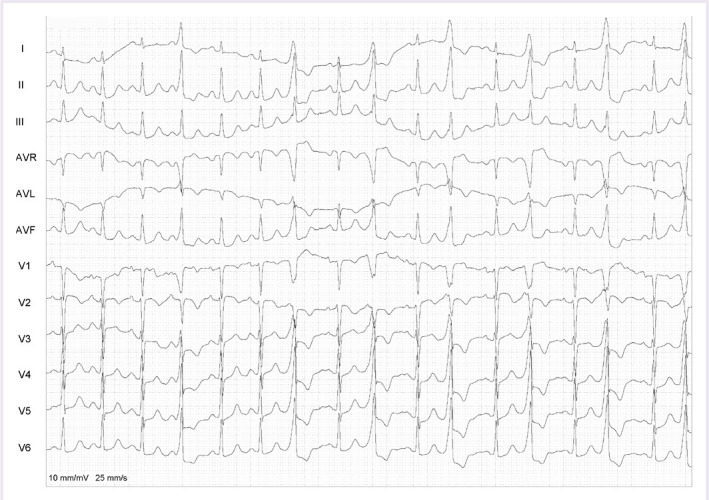
Post-ablation 12-lead surface ECG. ECG: Electrocardiogram.

It was decided not to perform ablation at the region with the largest His potential due to the AV block risk. RF ablation was planned to the region, where the His potential amplitude was lower (0.2 mV), the AV ratio was <1, and ventricular activation preceded the QRS onset by 37 ms (Fig. 3). Subsequently, irrigated RF current was delivered using a steerable sheath (Agilis, St Jude Medical) in the distal his region with power starting at 15W with an irrigation flow rate of 10 ml/min after PVC was suppressed, RF delivery was applied for a total of 90 s with gradually increasing power to 25 W with an irrigation flow rate of 15 ml/min with maximum temperature of 42°C (Fig. 4a, b). There was no junctional beat during the RF energy application. After ablation, under intravenous isoproterenol infusion (4 mcgr/dk) and burst pacing from the right ventricle no PVCs/VTs were observed during the waiting period of 30 min (Fig. 5). After a 3 month follow-up period, the patients was asymptomatic and a 24-h ambulatory Holter recording performed. Ambulatory monitoring showed a PVC burden 0.1% without any antiarrhythmic agent.

## DISCUSSION

Here, we reported a case of successful catheter ablation of PVCs arising from the para-hisian region with a 3D electroanatomic mapping system. The study by Yamauchi et al. [4] demonstrated that para-hisian PVCs/VT had distinctive features on 12-lead ECG. The study reported that para-hisian PVCs/VT had electrocardiographic characteristics such as monophasic tall R wave present in lead I, relatively small R wave in lead III than in lead II, R wave present in lead avL, a relatively narrow QRS duration in the inferior leads, a QS pattern in lead V1, an early precordial transitional zone in leads V2-V3 and a relatively tall R wave in V5, V6. In a similar vein, the clinical PVC morphology was consistent with para-hisian origin in our case. Ablation in this region could be challenging due to potential damage to AV conduction. Mapping and ablation could cover all the structures adjacent to the His-bundle region including: (a) RV septum underneath the tricuspid valve (TV); (b) right coronary cusp (RCC) or non-coronary cusp (NCC); (c) left ventricular (LV) septum below the aortic valve; and (d) the contiguous right atrium (RA). The study by Candemir et al. [5] reported a successful RF catheter ablation of para-hisian PVCs at the RV septum, underneath TV, using a “reverse S-curve” approach. The previous studies demonstrated successful RF catheter ablations of para-hisian PVCs from aortic sinus cusps (RCC, and NCC) [6, 7]. The distance from the endocardial LV surface to the His-bundle is shorter than from the right site, making the His more vulnerable when mapping the basal LV septum. Therefore, there is a lower likelihood of success and a higher AV block risk in RF catheter ablations [8]. To mapping of the contiguous RA, the ablation catheter is positioned at the inferior and medial aspect of the RA. In this area, a small atrial signal and a larger ventricular signal is usually recorded. Studies have shown an association between high PVC burden and LV systolic dysfunction. Baman et al. [9] reported that a PVC burden of >24% was an independently associated with PVC-induced cardiomyopathy. Moreover, in a consecutive series of 294 subjects with frequent PVCs, independent predictors of PVC-induced cardiomyopathy were PVC burden, QRS duration, epicardial origin, and symptom duration [10]. PVC ablation was performed for the reason that PVC burden was 25.3% and the PVC were persistent under medical treatment in our patient. We performed the RF catheter ablation using the “the contiguous RA” approach. A successful RF catheter ablation was done in the para-hisian region that small atrial signal and distal His potential were observed and ventricular activation preceded the QRS onset by 37 ms using electroanatomic mapping system. Although it is appropriate to consider cryoablation, especially in para-hisian PVCs resistant to medical treatment such as ours, we choose to use RF energy due to its versatile approach for mapping and ablation of all the structures adjacent to the His-bundle region and lower rate of arrhythmia recurrence. We consider that; applying the gradual RF energy, performing RF energy at the distance as far as possible from the largest His potential and delivering RF energy on the distal His area covered with a central fibrous body reduced the risk of AV block.

## Conclusion

We performed RF catheter ablation of PVCs originating from the para-hisian region using a 3D electroanatomic mapping system. The gradual RF energy delivery and the detailed activation mapping along with the distance from the largest His-bundle potential decreased the AV block risk. We believe that RF catheter ablation treatment, which will be applied to the high-burden PVC origin with a limited response to pharmacotherapy, can be also preferred in the para-hisian regions.
